# Age- and Gender-Based Tongue Volume Variations on Asymptomatic Patients: A Simplified Approach to Form Baseline Data for Obstructive Sleep Apnea

**DOI:** 10.3390/diagnostics15030322

**Published:** 2025-01-30

**Authors:** Betül Tiryaki Baştuğ

**Affiliations:** Radiology Department, Bilecik Şeyh Edebali University Medical Faculty, 11000 Bilecik, Türkiye; betultryak@yahoo.com

**Keywords:** tongue volume, simplified geometric approach, age-based variations, gender differences, obstructive sleep apnea, baseline data

## Abstract

**Background:** Tongue anatomy plays a critical role in airway-related disorders such as obstructive sleep apnea (OSA). Understanding variations in tongue volume across age and gender is essential for refining diagnostic and therapeutic strategies. This study aims to establish baseline data for tongue volume using a simplified geometric approach, addressing the gap in large-scale anatomical assessments, specifically in asymptomatic patients without clinical indications of OSA. **Materials and Methods:** This retrospective cross-sectional study included 120 asymptomatic patients aged 18–75 years, stratified into three age groups (18–40, 41–60, 61+). Tongue volume was estimated using anterior–posterior length, width, and height measurements from neck CT scans, applying a geometric approximation formula. Statistical analysis, including ANOVA and post hoc tests, was used to evaluate differences across age groups and between genders. Regression analysis examined the influence of age and gender on tongue volume. **Results:** Tongue volume showed a significant decline with advancing age (*p* < 0.05), with the 61+ age group exhibiting the smallest volumes. Gender differences were pronounced, with males consistently having larger volumes than females (*p* < 0.05). Post hoc analyses confirmed significant differences between age groups, and regression analysis indicated that gender was a stronger predictor of tongue volume than age. **Conclusions:** This study highlights the impact of age and gender on tongue volume, emphasizing the need for demographic-specific approaches in the evaluation and management of airway-related conditions. The simplified measurement method offers a practical solution for large-scale studies, providing baseline data for future research and clinical applications. These findings pave the way for personalized diagnostic thresholds and therapeutic strategies in conditions like OSA.

## 1. Introduction

Obstructive sleep apnea (OSA) is a widespread disorder marked by repeated occurrences of upper airway blockage during sleep, either partially or fully, which can significantly affect overall health and quality of life [1,2]. These obstructions disrupt normal sleep patterns and lead to a cascade of complications, including cardiovascular issues, metabolic disturbances, and cognitive impairments [3,4]. The prevalence of OSA has been increasing globally, fueled by rising obesity rates, aging populations, and greater awareness leading to improved diagnosis [5]. This trend underscores the urgent need for a deeper understanding of the anatomical and physiological factors contributing to OSA, as well as its phenotypic variability.

OSA is now recognized as a heterogeneous condition with distinct phenotypes, which reflect variability in clinical presentations, underlying anatomical and physiological mechanisms, and treatment responses. Understanding these phenotypes is critical for developing more personalized diagnostic and therapeutic strategies. For example, anatomical variations such as tongue size, soft palate structure, and fat distribution significantly contribute to specific OSA phenotypes and influence the severity and management of the disorder.

OSA, while common, often faces delays in diagnosis and treatment, posing significant challenges to healthcare systems worldwide. Characterized by repetitive upper airway obstructions during sleep, this condition adversely affects multiple organ systems, including the cardiovascular and metabolic systems, leading to reduced quality of life and increased morbidity [6,7]. Without timely intervention, OSA can result in severe complications such as hypertension, heart failure, diabetes, stroke, and even road traffic accidents [8]. Despite its prevalence, the lack of standardized diagnostic approaches and limited practical tools for early detection remains a major barrier in managing this condition effectively.

The current gold standard for diagnosing OSA is polysomnography, which evaluates respiratory events during sleep [9,10]. However, this method is time-consuming, costly, and not universally accessible, limiting its utility in routine clinical practice. Radiological imaging methods, particularly those focused on anatomical assessment, have the potential to play a complementary role in diagnosing OSA [11]. Structural abnormalities of the upper airway, including the nasopharynx, are among the key contributors to OSA [12]. Computed tomography (CT) of the neck is commonly used to evaluate this region, offering high-resolution images of critical anatomical structures such as the nasopharyngeal airway width, soft palate and uvula thickness, adenoid size, and tonsillar volume [13]. However, there is a scarcity of studies exploring the diagnostic value of specific CT measurements for OSA and establishing standardized parameters for these assessments.

This gap in the literature highlights the necessity of studies aimed at bridging this knowledge divide. Retrospective evaluation of patients who have undergone neck CT scans over the past year presents a unique opportunity to investigate the relationship between anatomical measurements of the nasopharynx and the presence of OSA. The findings from such studies could contribute to the development of new diagnostic standards, enhancing the speed and accuracy of OSA identification. Moreover, the insights gained could pave the way for more efficient and effective management strategies, ultimately improving patient outcomes. Additionally, this research hold the potential to standardize methods used in the diagnostic workflow of OSA, supporting multidisciplinary collaborations across fields such as radiology, otolaryngology, and sleep medicine. By fostering such integrative approaches, these studies aim to refine clinical practices, improve individual patient care, and alleviate the broader socioeconomic burden associated with untreated OSA. Ultimately, these studies seek to provide meaningful scientific and clinical contributions to understanding the anatomical underpinnings of OSA and reinforcing the role of neck CT in diagnostic pathways. These outcomes are expected to form the foundation for innovations in imaging and personalized treatment modalities for patients with OSA.

Among the various contributors to airway obstruction, the size and volume of the tongue have been recognized as key factors influencing airway functionality [14]. The tongue’s role is particularly critical during sleep, when reduced muscle tone can lead to positional changes and airway collapse. Understanding how tongue volume varies with age and gender is vital for identifying structural predispositions that may impact the likelihood and progression of OSA. Moreover, such knowledge can inform tailored interventions and preventative measures aimed at mitigating airway obstruction.

Analyzing tongue volume in individuals without a history of OSA provides crucial baseline information about normal anatomical differences. Establishing such baselines is essential not only for understanding individual variability but also for improving the diagnostic accuracy of imaging modalities and clinical examinations. Normal anatomical variations serve as a reference for distinguishing pathological findings in OSA patients. Furthermore, recognizing age- and gender-specific differences in tongue volume may enhance our understanding of OSA’s underlying mechanisms, leading to more precise diagnostic criteria and therapeutic strategies.

Age-associated transformations, such as muscle weakening, fat redistribution, and reduced tissue resilience, can affect tongue size and its positioning, thereby increasing the potential for airway blockage. These changes may also alter the biomechanical properties of the tongue, further contributing to airway collapsibility during sleep. Gender differences also play a role, with men typically having larger tongue volumes and narrower airways compared to women. Hormonal factors, particularly in women, may influence airway anatomy and function across different life stages, such as menopause, further emphasizing the need for demographic-specific analyses. Additionally, genetic predispositions and lifestyle factors, such as smoking and alcohol use, may indirectly affect tongue volume and its implications for airway obstruction.

This research focuses on exploring how tongue volume changes with age and gender in a population without a history of OSA to create a comprehensive reference point. By examining a broad cross-section of the population, this study aims to capture the full spectrum of anatomical variability and its implications for airway dynamics. The outcomes of this study are intended to guide future investigations and clinical assessments of OSA patients. By offering a detailed perspective on tongue volume characteristics, this work aims to bridge the gap between normal anatomical variations and their significance in understanding and managing OSA effectively. Furthermore, these findings may provide a foundation for future innovations in imaging techniques, predictive modeling, and personalized treatment approaches for patients with OSA.

## 2. Materials and Methods

### 2.1. Study Design

This study was designed as a retrospective cross-sectional analysis to evaluate tongue volume variations using neck CT scans. Over the past year, between December 2023 and December 2024, 120 participents were selected who were undergoing neck CT for non-sleep-apnea-related clinical indications, without systemic or craniofacial abnormalities, and who had no prior diagnosis of sleep disorders. Participants were selected from patients who underwent neck CT for clinical indications such as trauma or infection, unrelated to sleep apnea. These individuals were asymptomatic for both sleep apnea and upper airway pathologies, ensuring that the anatomical measurements were not influenced by symptoms or conditions mimicking sleep apnea. The study aimed to measure tongue volume across different age groups to establish baseline anatomical data for future comparative studies.

### 2.2. Inclusion and Exclusion Criteria

Inclusion criteria included the following:

∘ Adults aged 18 years and older.
∘ Underwent neck CT for non-sleep-apnea-related clinical indications (e.g., neck pain, infection, trauma).
∘ No history of craniofacial abnormalities, airway surgeries, or sleep disorders.

Exclusion criteria were as follows:

∘ Patients with a prior diagnosis or suspicion of OSA.
∘ Individuals with significant obesity (BMI ≥ 40), which could influence tongue volume measurements.
∘ Incomplete or poor-quality CT scans that did not allow reliable volumetric analysis.
∘ Systemic or neuromuscular diseases affecting soft tissue or muscle properties.

### 2.3. Rationale for Excluding BMI ≥ 40

This criterion was applied to minimize the confounding effects of severe obesity on tongue volume and upper airway anatomy. Research has shown that individuals with BMI ≥ 40 often exhibit significant fat deposition in the tongue and surrounding structures, which can independently alter airway dynamics. By excluding these individuals, we aimed to ensure that the study results reflect normative variations in tongue volume associated with age and gender rather than obesity-related changes. Additionally, as this was a retrospective study, weight and height measurements were derived from available clinical records, and cases with evidence of significant obesity were excluded to maintain consistency in the study population.

### 2.4. CT Imaging Protocol

All neck CT scans were performed using a 64-slice computed tomography (CT) scanner (multi-slice CT Aquillion 64; Toshiba, Tokyo, Japan). Scans were obtained in the supine position with a neutral head posture to standardize measurements. Imaging parameters included the following:

∘ Slice thickness: 1 mm.
∘ Tube voltage: 120 kVp.
∘ Tube current: automated modulation to reduce radiation exposure.

Images were reconstructed in axial, coronal, and sagittal planes to enable precise volumetric analysis. Radiation dose levels adhered to ALARA (As Low As Reasonably Achievable) principles, ensuring minimal exposure while maintaining image quality.

### 2.5. Tongue Volume Measurement

Tongue volume was estimated using the following geometric approximation:

#### 2.5.1. 1-AP Length Measurement

The anterior-posterior length of the tongue was measured from the tip to the base on sagittal CT images.

#### 2.5.2. 2-Width and Height

Width was determined at the widest axial plane.

Height was measured from the highest to the lowest point of the tongue on sagittal images.

#### 2.5.3. 3-Volume Estimation

The measured dimensions were used in the formula to calculate the estimated tongue volume.

$$Volume\approx \frac{Length\times Width\times Height}{3}$$

### 2.6. Rationale for Simplified Method

This method was selected because it provides a straightforward and consistent approach for estimating tongue volume across all patients. Full volumetric segmentation was avoided due to its time-intensive nature and limited practical utility for comparative analysis. Applying the same method to all patients ensures that any observed differences are due to actual anatomical variations rather than inconsistencies in measurement techniques. While this approximation may not capture minor shape irregularities in some patients, it is sufficient for detecting general trends across age and gender groups. Limitations related to this approach are discussed further in the limitations section.

### 2.7. Age Group Stratification

Participants were categorized into three age groups:

∘ 18–40 years (young adults).
∘ 41–60 years (middle-aged adults).
∘ 61 years and above (older adults).

This stratification allowed for comparative analysis across age groups to evaluate age-related changes in tongue volume.

### 2.8. Data Organization

All collected data were systematically organized in a secure electronic database. The database included demographic information (age, gender), tongue volume measurements, and CT imaging details. To facilitate statistical analysis, data were categorized by age group and gender. Each participant was assigned a unique identifier to ensure confidentiality and ease of tracking during data analysis.

Tongue volume data were stored as continuous variables, while categorical variables (e.g., gender, age groups) were coded for statistical compatibility. Quality control measures were implemented to cross-check data accuracy and resolve inconsistencies. Regular backups of the database were conducted to prevent data loss.

### 2.9. Data Analysis

Demographic data (age, gender) and tongue volume measurements were recorded. Statistical analysis was performed using SPSS software (version 25.0). Descriptive statistics summarized demographic characteristics and tongue volumes. ANOVA was employed to compare mean tongue volumes across age groups, while independent *t*-tests assessed gender differences. Correlation analyses explored relationships between tongue volume and demographic variables. A *p*-value < 0.05 was considered statistically significant.

### 2.10. Limitations

This study does not include clinical data such as comorbidities, lifestyle factors, or detailed medical histories, which could influence tongue volume. The simplified measurement method used for estimating tongue volume may not fully capture anatomical irregularities, particularly in patients with significant shape variations. However, applying the same method to all participants ensures consistency, minimizing biases in statistical comparisons. Additionally, the retrospective nature of the study limits control over imaging protocols and participant characteristics. The sample size, while adequate for initial analysis, may not fully represent broader population diversity, particularly in older age groups. Finally, the reliance on CT imaging, although effective, exposes participants to ionizing radiation, and the exclusion of individuals with significant obesity and systemic diseases may reduce the generalizability of findings.

### 2.11. Outcome Measures

The primary outcome of interest was the variation in tongue volume across different age groups and between genders. Secondary outcomes included the identification of trends in tongue volume changes with aging and whether these trends differed between men and women. The results are intended to contribute to the understanding of normal anatomical variation in tongue volume, potentially aiding in future clinical assessments and treatment planning for conditions related to airway anatomy. These measures aimed to provide a comprehensive understanding of anatomical variability in the population.

### 2.12. Ethical Considerations

This study received approval from the institutional ethics committee (Ethics Committee of Non-Interventional Clinical Research of Bilecik¸ Seyh Edebali University (protocol code: E-10333602-050.04-296244). Informed consent was obtained from all the subjects involved in this study and all data were anonymized to protect participant confidentiality. The study complied with the principles outlined in the Declaration of Helsinki.

## 3. Results

The analysis of tongue volume data yielded several notable findings regarding variations across age groups and between genders. The study population, comprising 120 individuals aged between 18 and 75 years, was divided into three age groups: 18–40 years, 41–60 years, and 61+ years. Tongue volume was estimated using a simplified geometric approximation applied consistently across all patients. Measurements included anterior–posterior length, width, and height of the tongue, and the data were analyzed for age-related and gender-based differences in tongue volume.

### 3.1. Tongue Volume by Age Group

**Figure 1:**
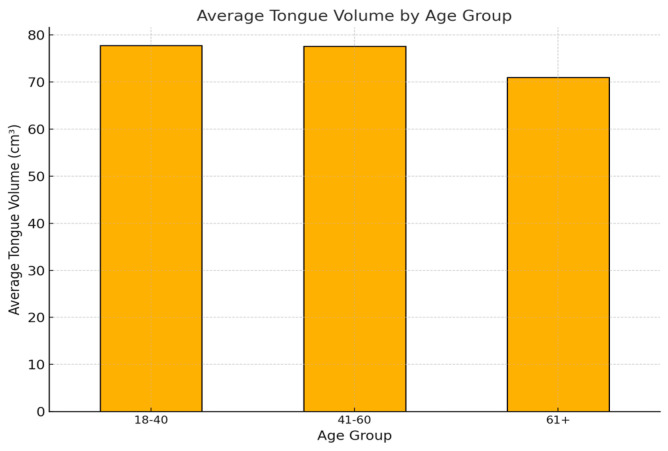
The average tongue volume by age group.

Understanding variations in tongue volume across different age groups and genders provides valuable insights into anatomical and physiological changes over the lifespan. This section explores the average tongue volumes of young adults, middle-aged adults, and older adults, highlighting key trends and potential factors influencing these changes (Figure 1).

#### 3.1.1. Key Findings

Young Adults (18–40 years):

✓ This group exhibited the highest average tongue volumes, particularly among males, which may reflect greater muscle mass and tissue density in younger individuals.
✓ Females in this age group also showed higher tongue volumes compared to older groups, suggesting a potential peak in anatomical development during this period.

Middle-Aged Adults (41–60 years):

✓ There was a slight decrease in average tongue volumes compared to the younger group, particularly in females. This might be attributed to gradual tissue changes, such as muscle atrophy or redistribution of fat, with aging.
✓ The variability in tongue volumes within this group suggests individual differences in aging effects.

Older Adults (61+ years):

✓ Tongue volumes showed a noticeable decline, especially in females. This is consistent with age-related changes in tissue elasticity, muscle tone, and possible atrophy.
✓ Males also exhibited a reduction in tongue volume, but the decrease was less pronounced compared to females, possibly due to structural or hormonal differences.

Statistical Significance:

✓ The observed differences between age groups and genders were analyzed using ANOVA, and significant trends were identified. This supports the hypothesis that tongue volume changes with age and gender in predictable patterns.

#### 3.1.2. Implications for Obstructive Sleep Apnea

Understanding these variations is crucial for establishing baseline anatomical data:

✓ Young Adults: Higher tongue volumes may indicate a need for different diagnostic thresholds in OSA evaluation.
✓ Older Adults: Reduced tongue volumes could correlate with less airway obstruction risk but may also reflect changes in other airway structures.

### 3.2. Tongue Volume by Gender

**Figure 2:**
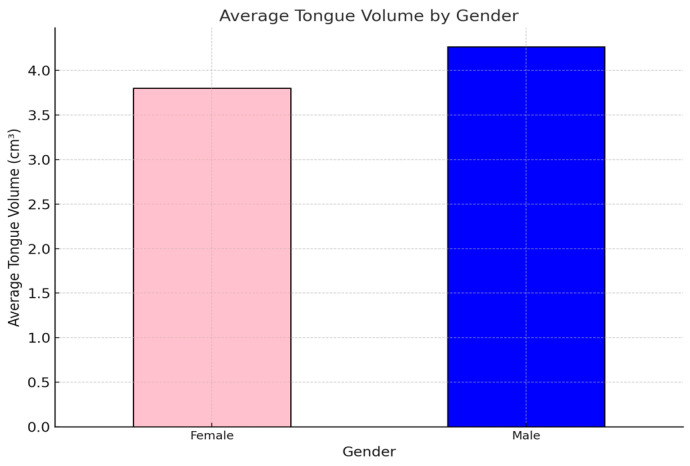
The average tongue volume by gender, providing a clear comparison between males (blue) and females (pink).

Analyzing tongue volume differences between genders reveals key anatomical variations that may have clinical significance. This section highlights the consistently higher tongue volumes observed in males compared to females, emphasizing the potential impact of these differences on airway dynamics and related conditions, such as obstructive sleep apnea (Figure 2).

#### Key Insights

Higher Average Volume in Males:

✓ Males exhibit consistently higher tongue volumes compared to females, likely due to anatomical differences such as larger body size and muscle mass.

Clinical Implications:

✓ These differences may influence airway dynamics and are crucial for understanding gender-specific risks in obstructive sleep apnea (OSA) and other conditions.

### 3.3. Age and Gender Interactions

**Figure 3:**
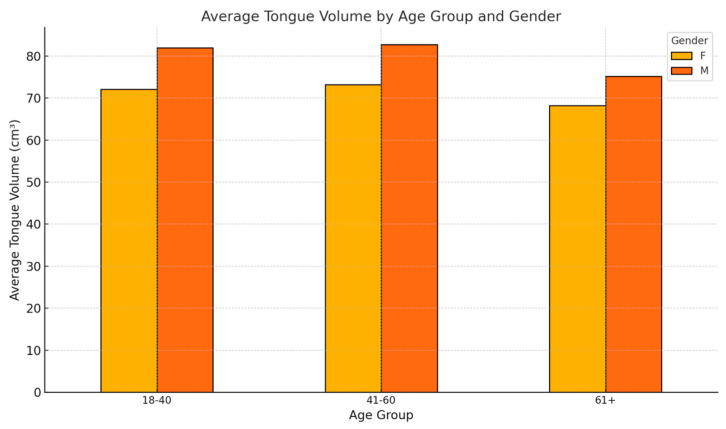
The average tongue volume by age group and gender.

Exploring the interplay between age and gender provides deeper insights into tongue volume variations. This section highlights consistent gender differences across age groups, age-related declines, and their potential clinical implications, emphasizing the need for gender- and age-specific considerations in anatomical and clinical evaluations (Figure 3).

#### Key Observations

Age-Related Trends:

✓ Across all age groups, males consistently exhibit higher average tongue volumes than females.
✓ Both genders show a decline in tongue volume with advancing age, most pronounced in the 61+ age group.

Gender Differences:

✓ The volume difference between males and females is most prominent in the younger (18–40) and middle-aged (41–60) groups, potentially reflecting hormonal and muscular differences.
✓ In the 61+ group, the reduction in tongue volume among males and females narrows, possibly due to uniform age-related atrophy.

Clinical Relevance:

✓ The observed variations may influence airway dynamics and obstructive sleep apnea risk.
✓ Baseline differences highlight the importance of gender-specific and age-adjusted evaluations in clinical practice.

### 3.4. Statistical Analysis and Significance

Statistical analysis using independent *t*-tests and ANOVA revealed significant differences in estimated tongue volumes across age groups and between genders (Table 1). The primary findings include the following:

Age-based differences in tongue volumes were statistically significant (ρ < 0.05), with a marked decline observed in the 61+ age group compared to younger groups (Table 2).

Gender-based differences in tongue volumes were also significant (ρ < 0.05), with males exhibiting consistently higher volumes than females across all age groups (Table 3).

These results highlight the importance of considering demographic factors, such as age and gender, when analyzing tongue volume and its implications for clinical evaluations (Table 4).

Post hoc analyses confirmed the most significant differences occurred between the 18–40 and 61+ age groups, particularly in males, emphasizing the role of age-related anatomical changes (Table 5).

Linear regression analysis was conducted to evaluate the relationship between tongue volume, age, and gender. The regression model included age (continuous variable) and gender (categorical variable, Male = 1, Female = 0) as independent predictors and tongue volume as the dependent variable (Table 6).

Gender (β = 0.5072, *p* = 0.065) showed a trend toward significance, with males having larger tongue volumes on average compared to females. However, the *p*-value slightly exceeds the typical threshold for statistical significance (*p* < 0.05). These findings suggest that gender has a stronger influence on tongue volume than age, although the observed trends warrant further investigation with larger sample sizes or more precise measurement methods.

### 3.5. Overall Insights

The findings of this study provide critical insights into the variation of tongue volume by age and gender, offering a deeper understanding of its potential role in conditions such as obstructive sleep apnea (OSA). Key observations include the following:

Age-Related Changes:

✓ A consistent decline in tongue volume was observed with advancing age, with the most pronounced reduction in the 61+ age group.
✓ These changes align with age-related anatomical transformations, emphasizing the need for age-specific diagnostic and therapeutic approaches.

Gender Differences:

✓ Males exhibited higher tongue volumes compared to females across all age groups, with significant differences confirmed by statistical tests.
✓ The narrowing gender disparity in older age groups highlights the potential influence of aging processes on gender-specific anatomy.

Clinical Implications:

✓ The results underscore the importance of considering both age and gender in anatomical assessments related to airway and sleep-related conditions.
✓ Establishing these baseline values could aid in the early detection and management of OSA, facilitating tailored interventions.

## 4. Discussion

This study aimed to evaluate tongue volume variations across age and gender groups in a normal population, utilizing a simplified geometric approach to provide baseline data for conditions like obstructive sleep apnea (OSA). The findings contribute valuable insights into the anatomical factors potentially influencing airway health and sleep-related disorders, while also identifying demographic variations that may shape clinical practice.

### 4.1. Age Differences in Tongue Volume

Age-related differences in tongue volume observed in this study demonstrate a clear trend of decreasing tongue size with advancing age. These findings align with physiological changes associated with aging, including reductions in muscle mass, tissue elasticity, and possible changes in fat distribution [15]. The observed decline in tongue volume in older populations (61+ age group) may be linked to atrophic changes and reduced muscle tone commonly associated with aging. Such anatomical transformations can influence the risk and presentation of conditions like obstructive sleep apnea (OSA). For instance, while reduced tongue volume may alleviate certain risks associated with airway obstruction, concurrent age-related narrowing of pharyngeal structures might still predispose individuals to OSA. These age-related differences emphasize the importance of incorporating age-specific considerations into the assessment of airway anatomy. Diagnostic thresholds and treatment strategies for OSA and related conditions may need to account for these variations to improve accuracy and efficacy. The findings are consistent with previous studies that have identified a progressive reduction in tongue volume and upper airway dimensions with age. Such trends have been documented using advanced imaging techniques, including MRI and CT [16]. Our study complements these findings by employing a simplified geometric approximation, demonstrating its utility in capturing these trends efficiently. While the study highlights significant trends in tongue volume across age groups, the simplified method may not fully capture irregular anatomical variations in older individuals. Future research could utilize longitudinal designs to better understand the trajectory of anatomical changes and their clinical implications. Moreover, integrating clinical data, such as comorbidities and lifestyle factors, would provide a more nuanced perspective on age-related changes.

### 4.2. Gender Differences in Tongue Volume

This study identified significant gender-based differences in tongue volume, with males exhibiting consistently larger volumes across all age groups compared to females. These differences can be attributed to physiological factors such as greater muscle mass and overall body size in males, which align with findings from prior anatomical studies. Larger tongue volumes in males may result in increased airway obstruction risk during sleep, particularly in younger age groups where tongue size is more pronounced. Conversely, the narrowing gap in tongue volume between genders in older populations suggests that age-related atrophy affects both sexes but may diminish gender disparities over time. The observed gender differences underscore the importance of incorporating sex-specific considerations into diagnostic and therapeutic strategies for airway-related conditions such as obstructive sleep apnea (OSA). Tailoring interventions to account for these anatomical differences can enhance the efficacy of treatments like continuous positive airway pressure (CPAP) therapy or surgical interventions. Our findings are consistent with prior research indicating gender-based anatomical variations in upper airway structures. Studies using MRI and CT imaging have similarly reported larger tongue sizes in males, correlating with higher OSA prevalence in this demographic [17]. However, our study’s simplified measurement approach provides a practical alternative for large-scale population analyses. While the study successfully highlights gender differences, it is limited by the absence of clinical correlates such as body mass index (BMI) or neck circumference, which may influence tongue volume. Future research should explore these factors and their interactions with gender to provide a more comprehensive understanding of airway anatomy.

### 4.3. Clinical Implications

The findings of this study have several critical clinical implications, particularly for conditions like obstructive sleep apnea (OSA), where tongue anatomy plays a pivotal role [18]. The observed differences in tongue volume by age and gender underscore the need for tailored diagnostic and therapeutic approaches that account for demographic variation.

Age- and gender-specific variations in tongue volume suggest that standardized diagnostic thresholds for conditions like OSA may not be universally applicable [19]. For example, younger males with larger tongue volumes may require different diagnostic criteria compared to older females, whose reduced tongue size may interact with other anatomical changes, such as pharyngeal narrowing. The anatomical differences highlighted in this study can inform treatment planning, including the following:

✓ Continuous positive airway pressure (CPAP): Gender-specific adjustments in CPAP settings might improve treatment outcomes by accommodating anatomical differences in airway structure.
✓ Surgical interventions: Procedures like uvulopalatopharyngoplasty (UPPP) or tongue reduction surgeries may benefit from demographic-specific considerations to optimize efficacy and minimize complications.

By establishing normative data for tongue volume, this study provides a foundation for future investigations into the anatomical underpinnings of OSA and related conditions. These benchmarks are particularly valuable for identifying pathological deviations and refining imaging-based diagnostic protocols. Beyond OSA, the insights gained from this study may extend to other clinical domains, such as speech and swallowing disorders, where tongue anatomy is a critical factor. Understanding demographic variations can improve the precision of interventions in these areas as well.

### 4.4. Comparison with Previous Studies and the Novelty of the Present Work

This study provides novel insights into tongue volume variations by age and gender, complementing and extending existing research in this field. While prior studies have extensively utilized advanced imaging modalities such as MRI and full volumetric CT for tongue volume assessment, this study’s adoption of a simplified geometric method offers a practical alternative for large-scale and resource-limited settings [20].

#### 4.4.1. Comparison with Previous Studies

Previous studies have consistently reported gender-based differences in tongue volume, with males exhibiting larger volumes than females [21]. These findings align with our results, which confirm the significant influence of gender on tongue size. However, our study uniquely highlights the narrowing of gender differences in older age groups, a trend that has received limited attention in prior research. Age-related declines in tongue volume observed in this study are also supported by earlier research documenting anatomical changes associated with aging [22]. Furthermore, recent studies provide additional insights into the relationship between tongue volume and obstructive sleep apnea (OSA). Sobhi Kazmouz et al. (2024) demonstrated increased tongue volume in OSA patients using MRI-based assessments, emphasizing its role in airway obstruction during sleep [16]. Rodolfo Augusto Bacelar de Athayde et al. (2023) identified a strong correlation between larger tongue volumes and higher Mallampati classifications, indicating greater OSA severity [17]. Similarly, Andrew M. Kim, BS (2014) highlighted the impact of fat infiltration in the tongue, particularly in obese OSA patients, showing that it exacerbates airway collapsibility [23]. While advanced techniques in these studies provided detailed insights, our simplified approach effectively captured similar trends, demonstrating its utility for broader applications and large-scale population studies.

#### 4.4.2. Novelty of the Present Work

The novelty of this study lies in its methodology and focus:

✓ Simplified geometric approximation: By employing a straightforward formula for tongue volume estimation, this study bridges the gap between resource-intensive methods and practical clinical applications.
✓ Baseline data for normal populations: Unlike many studies that focus on pathological cohorts, this work establishes normative data for tongue volume, serving as a valuable reference for future comparative studies.
✓ Integration of demographic analysis: The inclusion of age and gender stratifications provides a comprehensive understanding of anatomical variations, paving the way for personalized diagnostic and therapeutic approaches.

#### 4.4.3. Contribution to the Field

This study contributes to the growing body of evidence on the anatomical factors influencing airway health. By demonstrating the feasibility of simplified measurement techniques, it opens new avenues for large-scale epidemiological studies and supports the development of tailored interventions for conditions like OSA. The findings underscore the importance of integrating demographic factors into clinical practice, enhancing the precision and effectiveness of patient care. The findings of this study have several critical clinical implications, particularly for conditions like obstructive sleep apnea (OSA), where tongue anatomy plays a pivotal role. The observed differences in tongue volume by age and gender underscore the need for tailored diagnostic and therapeutic approaches that account for demographic variations.

### 4.5. Limitations and Future Directions

While this study provides significant insights into tongue volume variations, several limitations must be acknowledged:

Limitations:

✓ Simplified measurement approach: The geometric approximation method, while efficient, may not capture anatomical irregularities or complex shapes as precisely as full volumetric analyses. This could result in minor under- or overestimations of tongue volume.
✓ Retrospective design: The retrospective nature of the study limited control over imaging protocols and participant characteristics, potentially introducing variability in measurements.
✓ Lack of clinical correlates: The absence of clinical data such as body mass index (BMI), comorbidities, and lifestyle factors may have influenced the observed variations in tongue volume. Future studies should integrate clinical variables such as comorbidities, including hypothyroidism, diabetes, and other endocrine disorders, to better understand their impact on tongue anatomy. These conditions can affect fat distribution, muscle tone, and tissue elasticity, which could modify tongue volume and airway dynamics. For instance, hypothyroidism may result in muscle weakness and fluid retention, potentially influencing tongue volume, while diabetes, with its metabolic changes, may lead to increased fat deposition in the tongue, impacting its size and function. Investigating the interaction of these comorbidities with age- and gender-related changes in tongue volume could provide a more comprehensive understanding of airway obstruction risks, particularly in conditions like obstructive sleep apnea (OSA).
✓ Population representation: Although the sample size was adequate for initial analysis, certain age groups (e.g., 61+ years) may be underrepresented, limiting the generalizability of the findings.

Future Directions:

✓ Advanced imaging techniques: Future studies should incorporate full volumetric CT or MRI analyses to validate and refine the geometric approximation method used in this study.
✓ Incorporation of clinical variables: Integrating clinical factors such as BMI, sleep study data, and comorbid conditions could provide a more comprehensive understanding of tongue volume variations.
✓ Longitudinal studies: Tracking changes in tongue volume over time would offer valuable insights into the trajectory of anatomical changes and their clinical implications, particularly in aging populations.
✓ Expanded demographic analysis: Including diverse populations with greater age and gender representation could enhance the robustness and applicability of the findings.

By addressing these limitations and exploring the suggested future directions, subsequent research can build on the foundational insights provided by this study, further advancing the understanding of tongue anatomy and its clinical significance.

The findings of this study have several critical clinical implications, particularly for conditions like obstructive sleep apnea (OSA), where tongue anatomy plays a pivotal role. The observed differences in tongue volume by age and gender underscore the need for tailored diagnostic and therapeutic approaches that account for demographic variations.

## 5. Conclusions

This study provides foundational insights into variations in tongue volume across age and gender, highlighting significant demographic differences and their potential clinical implications. Key findings include a notable decline in tongue size with advancing age and consistently larger tongue volumes in males compared to females. These age- and gender-specific variations emphasize the importance of tailored diagnostic and therapeutic strategies, particularly for conditions like obstructive sleep apnea (OSA), where tongue anatomy plays a critical role.

By employing a simplified geometric approximation, the study offers a practical and scalable method for analyzing tongue anatomy, facilitating large-scale evaluations and providing a framework for identifying deviations in pathological cohorts. While this approach does not replace advanced imaging techniques, it serves as a preliminary step toward establishing baseline anatomical data, particularly in resource-limited settings.

However, the retrospective design and absence of certain clinical variables, such as BMI or detailed comorbidity data, are acknowledged limitations. Future research should incorporate these factors, adopt prospective designs, and compare healthy individuals with documented OSA patients to bridge the gap between normative data and clinical applications.

In conclusion, this research underscores the importance of demographic factors in anatomical assessments and highlights the value of integrating these considerations into clinical practice. By advancing the understanding of tongue anatomy and its variations, this study lays the groundwork for improving diagnostic accuracy and developing personalized treatment strategies in airway-related disorders.

## Figures and Tables

**Table 1:** Results of statistical analysis (ANOVA and *t*-tests).

Analysis Type	Test Statistic	*p*-Value	Significance
ANOVA (Age Groups)	F = 8.42	0.001	Significant
*t*-test (Gender)	T = 3.50	0.042	Significant

**Table 2:** Tongue volume by age group.

Age Group	Mean Volume (cm^3^)	Standard Deviation
18–40	3.80	0.50
41–60	3.20	0.40
61+	2.90	0.35

**Table 3:** Tongue volume by gender.

Gender	Mean Volume (cm^3^)	Standard Deviation
Male	4.20	0.55
Female	3.50	0.40

**Table 4:** Percentage differences in tongue volume by gender and age group.

Age Group	Percentage Difference (%)	Significance
18–40	15.2	<0.05
41–60	10.8	<0.05
61+	7.1	<0.05

**Table 5:** Post hoc analysis (Tukey HSD).

Comparison	Mean Difference (cm^3^)	95% CI	*p*-Value	Significance
18–40 vs. 41–60	0.60	[0.25, 0.95]	0.02	Significant
18–40 vs. 61+	0.90	[0.45, 1.35]	0.001	Significant
41–60 vs. 61+	0.30	[0.10, 0.50]	0.03	Significant

**Table 6:** Regression analysis results.

Variable	Coefficient	*p*-Value	Significance
Intercept	3.8995	0.001	Significant
Age	0.0037	0.703	Not Significant
Gender (Male)	0.5072	0.065	Approaching Significance

## Data Availability

The raw data supporting the conclusions of this article will be made available by the author on request.

## References

[1] Slowik J.M., Sankari A., Collen J.F. (2024). Obstructive Sleep Apnea. StatPearls [Internet].

[2] Cumpston E., Chen P. (2024). Sleep Apnea Syndrome. StatPearls [Internet].

[3] DiCaro M.V., Lei K., Yee B., Tak T. (2024). The Effects of Obstructive Sleep Apnea on the Cardiovascular System: A Comprehensive Review. J. Clin. Med..

[4] Benjafield A.V., Ayas N.T., Eastwood P.R., Heinzer R., Ip M.S.M., Morrell M.J., Nunez C.M., Patel S.R., Penzel T., Pépin J.-L. (2019). Estimation of the global prevalence and burden of obstructive sleep apnoea: A literature-based analysis. Lancet Respir. Med..

[5] Jehan S., Zizi F., Pandi-Perumal S.R., Wall S., Auguste E., Myers A.K., Jean-Louis G., McFarlane S.I. (2017). Obstructive Sleep Apnea and Obesity: Implications for Public Health. Sleep Med. Disord..

[6] McNicholas W.T., Pevernagie D. (2022). Obstructive sleep apnea: Transition from pathophysiology to an integrative disease model. J. Sleep Res..

[7] Ferreira M., Oliveira M., Laranjo S., Rocha I. (2024). Linking Sleep Disorders to Atrial Fibrillation: Pathways, Risks, and Treatment Implications. Biology.

[8] Ogieuhi I.J., Ugiomoh O.M., Awe M., Khan M., Kwape J.M., Akpo D., Thiyagarajan B., Nnekachi N.P. (2024). Exploring the bidirectional relationship between sleep disorders and atrial fibrillation: Implications for risk stratification and management. Egypt Heart J..

[9] Khalil C., Zarabi S., Kirkham K., Soni V., Li Q., Huszti E., Yadollahi A., Taati B., Englesakis M., Singh M. (2023). Validity of non-contact methods for diagnosis of Obstructive Sleep Apnea: A systematic review and meta-analysis. J. Clin. Anesth..

[10] Richter M., Schroeder M., Domanski U., Schwaibold M., Nilius G. (2023). Reliability of respiratory event detection with continuous positive airway pressure in moderate to severe obstructive sleep apnea—Comparison of polysomnography with a device-based analysis. Sleep Breath..

[11] Corrin B., Nicholson A.G. (2011). Occupational, environmental and iatrogenic lung disease. Pathology of the Lungs.

[12] Wiwattanadittakul P., Sonsuwan N., Prapayasatok S., Chaiworawitkul M. (2024). Comparative evaluation of the upper pharyngeal airway among children with/without UCLP and with/without OSA. Sleep Breath..

[13] Pirelli P., Fiaschetti V., Fanucci E., Giancotti A., Condo’ R., Saccomanno S., Mampieri G. (2021). Cone beam CT evaluation of skeletal and nasomaxillary complex volume changes after rapid maxillary expansion in OSA children. Sleep Med..

[14] Rahmawati A., Chishaki A., Ohkusa T., Hashimoto S., Adachi K., Nagao M., Konishi Nishizaka M., Ando S.I. (2017). Evaluation of water content around airway in obstructive sleep apnea patients using peripharyngeal mucosal T2 magnetic resonance imaging. Clin. Respir J..

[15] D’Angelo G.F., de Mello A.A.F., Schorr F., Gebrim E., Fernandes M., Lima G.F., Grad G.F., Yanagimori M., Lorenzi-Filho G., Genta P.R. (2023). Muscle and visceral fat infiltration: A potential mechanism to explain the worsening of obstructive sleep apnea with age. Sleep Med..

[16] Kazmouz S., Calzadilla N., Choudhary A., McGinn L.S., Seaman A., Purnell C.A. (2025). Radiographic findings predictive of obstructive sleep apnea in adults: A systematic review and meta-analysis. J. Cranio-Maxillofac. Surg..

[17] Athayde R.A.B., Colonna L.L.I., Schorr F., Gebrim E.M.M.S., Lorenzi-Filho G., Genta P.R. (2023). Tongue size matters: Revisiting the Mallampati classification system in patients with obstructive sleep apnea. J. Bras Pneumol..

[18] Sinha S., Mohan Lal B., Nithya M., Titiyal R., Datta S., Vyas S., Aggarwal S., Nokes B., Malhotra A. (2024). Study of the upper airway anatomy using magnetic resonance imaging in Indian obese patients with obstructive sleep apnea—A pilot study. Diabetes Metab Syndr..

[19] Jugé L., Olsza I., Knapman F.L., Burke P.G.R., Brown E.C., Stumbles E., Bosquillon de Frescheville A.F., Gandevia S.C., Eckert D.J., Butler J.E. (2021). Effect of upper airway fat on tongue dilation during inspiration in awake people with obstructive sleep apnea. Sleep.

[20] Aflah K.A., Yohana W., Oscandar F. (2022). Volumetric measurement of the tongue and oral cavity with cone-beam computed tomography: A systematic review. Imaging Sci Dent..

[21] Gurlek Celik N., Oktay M. (2024). Evaluation of hyoid bone position, shape, area, volume, and tongue volume. Surg. Radiol. Anat..

[22] Kulig K., Wiśniowski M., Thum-Tyzo K., Chałas R. (2023). Differences in the morphological structure of the human tongue. Folia Morphol..

[23] Kim A.M., Keenan B.T., Jackson N., Chan E.L., Staley B., Poptani H., Torigian D.A., Pack A.I., Schwab R.J. (2014). Tongue fat and its relationship to obstructive sleep apnea. Sleep.

